# MCPeSe: Monte Carlo penalty selection for graphical lasso

**DOI:** 10.1093/bioinformatics/btaa734

**Published:** 2020-08-17

**Authors:** Markku Kuismin, Mikko J Sillanpää

**Affiliations:** Research Unit of Mathematical Sciences, University of Oulu, Oulu FI-90014, Finland; Biocenter Oulu, University of Oulu, Oulu FI-90014, Finland; Research Unit of Mathematical Sciences, University of Oulu, Oulu FI-90014, Finland; Biocenter Oulu, University of Oulu, Oulu FI-90014, Finland; Infotech Oulu, University of Oulu, Oulu FI-90014, Finland

## Abstract

**Motivation:**

Graphical lasso (Glasso) is a widely used tool for identifying gene regulatory networks in systems biology. However, its computational efficiency depends on the choice of regularization parameter (tuning parameter), and selecting this parameter can be highly time consuming. Although fully Bayesian implementations of Glasso alleviate this problem somewhat by specifying *a priori* distribution for the parameter, these approaches lack the scalability of their frequentist counterparts.

**Results:**

Here, we present a new Monte Carlo Penalty Selection method (MCPeSe), a computationally efficient approach to regularization parameter selection for Glasso. MCPeSe combines the scalability and low computational cost of the frequentist Glasso with the ability to automatically choose the regularization by Bayesian Glasso modeling. MCPeSe provides a state-of-the-art ‘tuning-free’ model selection criterion for Glasso and allows exploration of the posterior probability distribution of the tuning parameter.

**Availability and implementation:**

R source code of MCPeSe, a step by step example showing how to apply MCPeSe and a collection of scripts used to prepare the material in this article are publicly available at GitHub under GPL (https://github.com/markkukuismin/MCPeSe/).

**Supplementary information:**

Supplementary data are available at *Bioinformatics* online.

## 1 Introduction

The graphical lasso (Glasso) (Banerjee *et al.*, 2008; Friedman *et al.*, 2008) is one of the most popular tools for Gaussian graphical model (GGM) selection: the papers of Friedman *et al.* (2008) and Banerjee *et al.* (2008) describing its use have been cited over 1821 and 544 times, respectively (Web of Science database, May 22, 2020). This is due to the following beneficial properties of *L*_1_ regularization: (i) the optimization of Glasso is a convex problem and thus has a reasonable computational cost, (ii) the estimates of the precision and covariance matrices obtained using Glasso are positive definite even though the corresponding maximum likelihood estimate is not and (iii) some of the off-diagonal elements in the precision matrix are suppressed exactly to zero, making it possible to use Glasso for GGM selection. Consequently, Glasso is a popular alternative to computationally intensive *L*_0_ regularization for penalized likelihood estimation.

However, the estimate computed with Glasso depends on the so-called tuning parameter (regularization parameter), which controls the sparsity of the selected GGM. Choosing this parameter is a challenging task. Some model selection criteria have been developed for selecting the Glasso tuning parameter. For example, Liu *et al.* (2010) introduced a stability approach to regularization selection (StARS) in Glasso models. StARS is based on subsampling of the data and selects the parameter such that it maximizes the stability of the undirected graph calculated based on these subsamples. Although this stability-based approach (see also Meinshausen and Bühlmann, 2010) is a general method for regularization parameter selection (cases involving continuous and discrete data), computing GGMs for each subsample is time consuming when the number of variables *P* is on the order of thousands. The extended Bayesian information criterion (eBIC) (Chen and Chen, 2008) is a more time-efficient method for regularization selection but it depends on a hyperparameter that controls the sparsity of the GGM and must be set manually. The Rotation Information Criterion (RIC) (Lysen, 2009; Zhao *et al.*, 2012) is an efficient tuning parameter selection method that scales to large datasets. Whereas StARS and eBIC depend on an extra tuning parameter, RIC can be considered a tuning-free method. For model selection in cases involving mixed data (i.e. a combination of continuous and discrete variables), see Lee and Hastie (2015) and Sedgewick *et al.* (2016).

Alternative Bayesian implementations of Glasso have also been proposed (Khondker *et al.*, 2013; Marlin and Murphy, 2009; Wang, 2012), but these do not scale to very high-dimensional problems (e.g. over 10k genes). Here, we focus on the Glasso model for continuous data.

To enable efficient tuning-free selection, we introduce a Monte Carlo penalty selection method (MCPeSe). This method uses the whole solution path computed with the frequentist Glasso to time-efficiently simulate the posterior distribution of the Glasso tuning parameter either using rejection sampling or the Metropolis–Hastings algorithm.

## 2 Examples

**Fig. 1.**
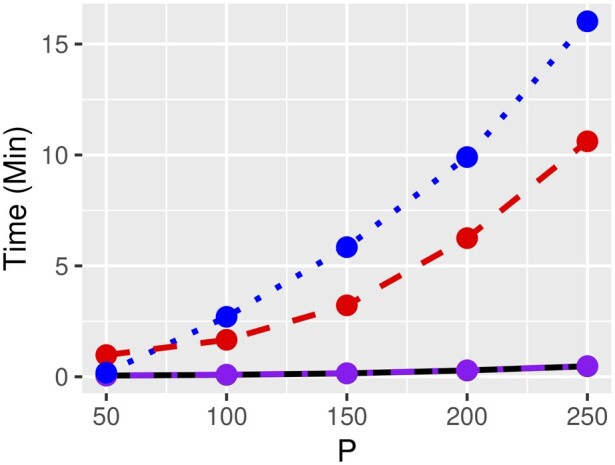
Computational times for MCPeSe (black solid line), Bayes Glasso (blue dotted line), RIC (purple dot-dashed line colliding with the black solid line) and StARS (red dashed line) as a function of *P*. A grid of 100 tuning parameter values was used for MCPeSe, StARS and RIC. 500k tuning parameter values were sampled with MCPeSe using rejection sampling. For Bayes Glasso, the number of burn-in iterations and length of Markov chain were both set to 100. With RIC, 20 rotations were computed. With StARS, 20 subsamples were drawn

We compare MCPeSe to eBIC, RIC and StARS and show that MCPeSe is a highly competitive tuning parameter selection method in terms of both computational time (Fig. 1) and graphical structure learning. In addition, in a binary classification test, the GGM determined with MCPeSe performed similarly to those determined with StARS and RIC in terms of sensitivity, precision and Matthews correlation coefficient. Further details can be found in the Supplementary Notes.

## 3 Implementation

The provided R implementation of MCPeSe is fully compatible with the widely used huge R package (Zhao *et al.*, 2012) (cited over 129 times according to the Web of Science database, May 22, 2020). The output of the function huge() from the huge package can be used as an input for the function mcpese().

The following code fragment shows how to run MCPeSe with huge:

# tuning parameter selection; data are provided as an *n *× *p* matrix

L = huge(Y, nlambda = 50, method=“glasso”)

MCPeSeSelect = mcpese(L, n=**nrow**(Y))

**names**(MCPeSeSelect)

“indx”“rhos”“accept.rate”“opt.rho”“opt.index”“n”

The function mcpese() returns the vector of indices of the selected tuning parameter values, simulated tuning parameter values, the accept rate, the smallest tuning parameter value greater than or equal to the mean of the estimated posterior distribution, the index of this tuning parameter and the sample size.

## 4 Conclusion

MCPeSe allows many different high-dimensional graphical models to be examined at little computational cost. In addition, the selected GGMs are comparable to those obtained using StARS and RIC. Combining MCPeSe with other network construction tools (see, e.g. Basu *et al.*, 2017) could thus facilitate the analysis of large-scale data.

For rapid dissemination and utilization of MCPeSe, an R implementation with detailed examples and descriptions of the method is available at GitHub and at *Bioinformatics* online.

## Supplementary Material

btaa734_Supplementary_Data
