# Supplementary material for: MCPeSe: Monte Carlo penalty selection for graphical lasso
Source: Bioinformatics. 2020 Aug 17;37(5):726–7. doi: 10.1093/bioinformatics/btaa734 (PMC8097680; doi:10.1093/bioinformatics/btaa734)
Supplement: btaa734_Supplementary_Data [file btaa734_supplementary_data.zip › SupplementaryMaterials.pdf]

MCPeSe: Monte Carlo penalty selection for graphical lasso - Supplementary materials

Markku Kuismin[1,2] and Mikko J. Sillanpää*[1,2,3]

[1]*Research Unit of Mathematical Sciences, University of Oulu, Finland*
[2]*Biocenter Oulu, University of Oulu, Finland*
[3]*Infotech Oulu, University of Oulu, Finland*

July 10, 2020

Abstract

Graphical lasso (Glasso) is a popular method for Gaussian graphical model (GGM) inference. The computational bottleneck of Glasso is its dependence on a regularization parameter (penalty parameter) whose selection can be very time-consuming and difficult. Fully Bayesian implementations of Glasso can alleviate the tuning parameter selection problem by specifying a prior distribution for this parameter. However, Bayesian implementations of Glasso usually lack scalability compared to their frequentist counterparts. Here we present Monte Carlo Penalty Selection (MCPeSe) for Glasso. MCPeSe combines the low computational cost of the frequentist Glasso with the ability to choose the tuning parameter using empirical Bayesian modeling. To our knowledge, MCPeSe is the fastest "automatic" model selection tool available for Glasso. The performance of MCPeSe in GGM inference is demonstrated using both simulated and empirical data sets. These examples show that MCPeSe is comparable to other tuning parameter selection tools such as eBIC, StARS and RIC. MCPeSe is available at GitHub (https://github.com/markkukuismin/MCPeSe) under the GPL license and is compatible with the `huge` R package.

Keywords: Gaussian graphical model, gene networks, graphical lasso, high-dimensional data, model selection, precision matrix, tuning parameter

1 Introduction

In many real-life research problems, it is practically impossible to experimentally examine dependencies between all variables of interest. Therefore, mathematical

*To whom correspondence should be addressed: mikko.sillanpaa@oulu.fi

and statistical modeling are used to gain insight into the original problem more cost-effectively. The development of fast optimization algorithms for maximizing the penalized likelihood of undirected Gaussian graphical models (GGMs) has made it possible to study interdependencies between variables very efficiently.

GGMs represent the conditional dependence relationships between random variables using a multivariate Gaussian distribution. In particular, two random variables are said to be independent given the remaining variables if the partial correlation between them is zero. GGMs are defined by setting specific partial correlation coefficients, or specific elements of the inverse covariance matrix (the precision matrix), to zero (Edwards, 2000). These models are commonly used to reconstruct gene regulatory networks from gene expression data, brain connectivity networks, networks in the field of psychology, and tumor cell networks among other things (see, e.g., Fitch and Jones, 2008; Krämer et al., 2009; Bühlmann et al., 2014; Epskamp and Fried, 2018; Petralia et al., 2018).

Unfortunately, research problems often have far more unknown parameters than data points. For example, a data set of riboflavin production by *Bacillus subtilis* (Lee et al., 2001; Zamboni et al., 2005; Bühlmann et al., 2014) contains 4088 unknown variables but only 71 samples. Throughout this manuscript, we use P and N to denote the number of random variables and samples respectively. When performing so-called high-dimensional statistics inference, it is commonly the case that $P \gg N$ and P is very large. It is not straightforward to apply maximum-likelihood (ML) estimation in GGM construction when dealing with high-dimensional data. For example, the ML estimate of the covariance matrix is not invertible, so the GGM cannot be determined. Therefore, various optimization-based likelihood methods (i.e., penalized log-likelihood maximization methods) have been proposed to infer high-dimensional GGMs.

L_0 penalized log-likelihood maximization is a straightforward way to obtain sparsity in the estimated precision matrix and has therefore drawn increasing interest in recent years (see, e.g., Wen et al., 2018, and references therein). However, optimization under L_0 regularization is very computationally expensive. Therefore, the less demanding L_1 penalty has become a popular alternative to L_0.

The L_1 penalty function introduces zeros into the precision matrix estimate and is therefore useful for undirected network reconstruction. More interest-

ingly, L_1 penalized log-likelihood maximization has enabled the study of high-dimensional data sets (Bühlmann and Van De Geer, 2011). In this article, we focus on the graphical lasso (hereafter Glasso) problem for continuous data (Banerjee et al., 2008; Friedman et al., 2008), in which the L_1 penalty function is used for ML estimation. In particular, we focus on the selection of the tuning parameter for Glasso.

Glasso has become a popular GGM selection method because it obviates the need to search the model space and allows the GGM to be constructed from the estimated precision matrix by adjusting a single parameter known as a tuning (penalty) parameter. However, choosing the tuning parameter for Glasso remains an open research question with no definite solution. Different model selection criterion have been proposed for continuous and discrete data based on extended Bayesian Information Criterion (eBIC) (Foygel and Drton, 2010), Stability selection (Liu et al., 2010; Meinshausen and Bühlmann, 2010), Rotation Information Criterion (RIC) (Lysen, 2009) and methods based on assumptions about the special structure of the ground truth network (e.g, Liu and Ihler, 2011; Li et al., 2018; Mestres et al., 2018). Model selection methods for mixed data (combination of continuous and discrete variables) have also been proposed (Lee and Hastie, 2015; Sedgewick et al., 2016).

Bayesian implementations of Glasso somewhat alleviate the regularization selection problem by treating the tuning parameter as a random variable and specifying a hyper-prior for it (Wang, 2012; Khondker et al., 2013). Despite the elegance of Bayesian modeling (which offers, at least in principle, a simple interpretation of how the prior is updated with the data to form a posterior distribution), Bayesian implementations present some practical problems in high-dimensional statistics. First, they are inferior to their frequentist counterparts in terms of time efficiency and scalability; generating a Markov chain Monte Carlo sample of a $P \times P$ precision matrix is very time-consuming. Second, the use of continuous prior distributions for precision matrix elements and the tuning parameter does not produce precision matrix posterior elements that shrink exactly to zero; consequently, some heuristic treatments (a separate cleaning step) are needed for GGM determination (Wang, 2012; Khondker et al., 2013). We note that other Bayesian models have been proposed for GGM selection that are not linked to penalized likelihood estimation (Carvalho and Scott, 2009;

Mohammadi and Wit, 2015; Williams et al., 2018).

The approach presented here shares the favorable properties of both (1) Bayesian modeling and (2) the "frequentist" algorithms developed to solve the Glasso problem time-efficiently. We show how the Glasso tuning parameter can be chosen based on a generated posterior sample of values using either the accept-reject (hereafter A-R) or the Metropolis-Hastings (hereafter M-H) style algorithms (see, e.g., Casella et al., 2004). We call our newly developed procedure MCPeSe: Monte Carlo Penalty Selection. MCPeSe is an empirical Bayes algorithm and to our knowledge, it is the fastest tuning-free way to choose tuning parameter value for Glasso. MCPeSe is an atypical implementation of a Markov Chain Monte Carlo method for this problem because instead of simulating values from a continuous candidate density we use a random sample from a discrete grid of pre-defined tuning parameter values.

Using simulations and empirical data sets, we show how GGMs selected with MCPeSe compare to those obtained using other model selection procedures such as eBIC, the Stability Approach for Regularization Selection (StARS) (Liu et al., 2010), and RIC.

2 Methods and Materials

2.1 Methods

We consider a model for continuous data in which the observations are assumed to have a multivariate Gaussian distribution. Let $\mathbf{Y} = (Y_1, \dots Y_P)^T$ be a P-dimensional random vector that follows a multivariate Gaussian distribution $N(\boldsymbol{\mu}, \Theta^{-1})$, where $\boldsymbol{\mu}$ is a $P \times 1$ mean vector and Θ^{-1} is a $P \times P$ inverse of the precision matrix. The precision matrix itself is the inverse of the covariance matrix, $\Sigma = \Theta^{-1}$. Without loss of generality, we assume that $\boldsymbol{\mu}$ is a zero vector. The GGM is defined by the undirected graph $G = (V, E)$, where $V = \{1, \dots, P\}$ is the set of nodes (vertices) and $E = V \times V$ is the set of edges. If two random variables Y_i and Y_j are conditionally independent given all other variables, the partial correlation coefficient between them is zero. This is equivalent to saying that the corresponding element of the precision matrix $\Theta = [\theta_{ij}]$ is zero. These zero (and non-zero) elements of the precision matrix uniquely code the sparsity

pattern of the graph G,

$$\theta_{ij} = 0 \Longleftrightarrow \{(i,j),(j,i)\} \notin E. \tag{1}$$

The problem is that the precision matrix is generally unknown, so the set E is also unknown. Several methods for estimating the precision matrix have been proposed (e.g., Meinshausen and Bühlmann, 2006; Friedman et al., 2008; Cai et al., 2011; Liu and Luo, 2015; Liu and Wang, 2017). In this article we use an estimate of the precision matrix computed with Glasso to select the undirected graphical model. Specifically, we use the "frequentist" Glasso algorithm instead of the Bayesian implementation to precalculate some quantities,

$$\underset{\Theta}{\operatorname{argmin}} \; -\log\det(\Theta) + \operatorname{tr}(S\Theta) + \rho||\Theta||_1, \tag{2}$$

where $\log(\cdot)$ is the natural logarithm, $\det(\cdot)$ is the matrix determinant, $\operatorname{tr}(\cdot)$ is the matrix trace, $S = \sum_{n=1}^{N} \mathbf{Y}_n \mathbf{Y}_n^\top / N$ is an estimator of the covariance matrix given a sample of N independent observations, ρ is a positive tuning parameter, and $||\Theta||_1 = \sum_{i \neq j} |\theta_{ij}|$ is the L_1 norm.

Following Wang (2012), the Glasso estimator is equivalent to the *maximum a posteriori* estimation of the following model:

$$\begin{aligned} p(\mathbf{Y}_n|\Theta) &\sim N(\mathbf{0}, \Theta^{-1}), \quad n = 1, \ldots N, \\ p(\Theta|\lambda) &\propto \prod_{i<j} \{De(\theta_{ij}|\lambda)\} \prod_{i=1}^{p} \{Exp(\theta_{ii}|\lambda/2)\} \, 1_{(\Theta>0)}, \end{aligned} \tag{3}$$

where $De(\cdot|\lambda)$ is the double exponential density function, $Exp(\cdot|\lambda)$ is the exponential density function, and $\lambda = N\rho$, and $1_{(\Theta>0)}$ is an indicator function of the event that the precision matrix Θ is positive definite. The tuning parameter (hyper-parameter λ) controls the sparsity pattern of the estimated precision matrix in (3) but choosing a suitable value for this parameter is not straightforward. Wang (2012) used a gamma prior for λ, giving a conditional gamma posterior distribution for λ.

We use the same hierarchical model as Wang (2012) but instead of fully relying on Markov chain Monte Carlo (MCMC) methods we want to utilize the frequentist Glasso because it is computationally much faster and more scalable than its Bayesian counterpart when facing problems with hundreds or thousands

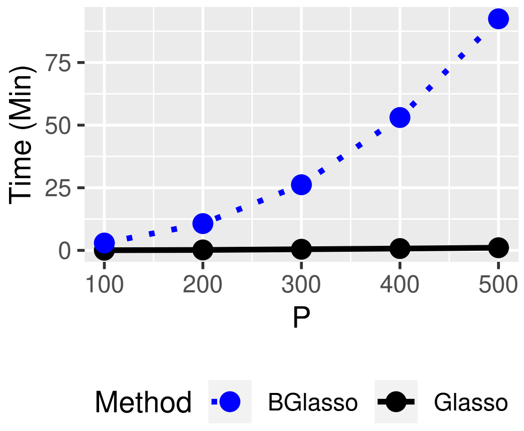

Figure 1: Computational times for Glasso and Bayesian Glasso (BGlasso) as a function of P. We used the `huge` and `BayesianGLasso` R packages to run Glasso (with $R = 50$ tuning parameter values) and BGlasso (200 MCMC steps), respectively.

of variables (see Fig 1). Therefore, from (3), where $\rho = \lambda/N$, we obtain the conditional posterior density of the tuning parameter ρ and the precision matrix Θ

$$\begin{aligned} p(\rho, \Theta|data) &\propto p(data|\Theta)p(\Theta|\rho)p(\rho) \\ &= L \times C^{-1}(N\frac{\rho}{2})^{\frac{P(P+1)}{2}} \exp{(-N\frac{\rho}{2}||\Theta||_1)} 1_{(\Theta>0)} \times p(\rho), \end{aligned} \quad (4)$$

where L is the likelihood function which depends on Θ, C is a normalizing constant, and $p(\rho)$ is the probability mass function of a uniform distribution abbreviated as $Unif[min(\rho), max(\rho)]$ or a gamma distribution restricted to the range $[min(\rho), max(\rho)]$ as in Wang (2012). Because we are using the frequentist Glasso to compute the precision matrix estimates $\widehat{\Theta}$ we can omit the indicator function in the above equation. For the same reason, our method is an empirical Bayesian method (see, e.g., Casella, 2001). This is because one dimension in our posterior distribution (4) is marginalized (instead of integrated) by maximization: we assume that $p(\rho|data) = \int p(\rho, \Theta|data)d\Theta \approx p(\rho, \widehat{\Theta}|data)$. Therefore, the resulting posterior will be somewhat too narrow because the uncertainty of Θ is not incorporated into the posterior of ρ.

We can restrict the support of our empirical prior distribution to the range

$[\min(|\widehat{r}_{ij}|), \max(|\widehat{r}_{ij}|)]$ because it has been shown that the reasonable upper and lower limits for the tuning parameter ρ for Glasso can be obtained from the absolute values of the MLE of the correlation matrix $R_Y = [r_{ij}]$ (Mazumder and Hastie, 2012; Basu et al., 2017; Fattahi and Sojoudi, 2019). When the data are standardized, running the Glasso algorithm with tuning parameter values smaller than the smallest absolute value of an off-diagonal element of the estimated correlation matrix yields a full graphical model in which each node of the network is a neighbor of other nodes. On the other hand, using a tuning parameter value greater than the largest absolute value of an off-diagonal element of the correlation matrix estimate will produce an empty graph with no edges. Therefore, sensible estimates of a sparse GGM can be found by inspecting the values of the tuning parameter ρ on the interval $[\min(|\widehat{r}_{ij}|), \max(|\widehat{r}_{ij}|)]$, $i, j = 1, \dots p$, $i \neq j$, where $\widehat{r}_{ij}$ is the estimated correlation coefficient between random variables i and j. We use these informative limits as *a priori* information on the tuning parameter, which saves considerable computational time when computing precision matrix estimates with Glasso. Additionally, it can be a lot faster not to run the Glasso with one tuning parameter value but to compute a full solution path of graphical models using a decreasing sequence of tuning parameter values (Friedman et al., 2010). Therefore, without advance knowledge about the optimal tuning parameter value, the most (time) efficient strategy is to compute a group of different graphical models and apply a suitable model selection criterion to select the best model among them. Next we describe how Monte Carlo methods can be used to estimate the posterior distribution for the tuning parameter using the solution path of a Glasso problem (2).

2.2 Tuning parameter posterior estimation and model selection with the accept-rejection method and the Metropolis-Hastings algorithm

After a reasonable grid of tuning parameter values has been set, one can construct as long a solution path of GGMs as one desires. One must then decide which graphical model to choose. We propose to apply either rejection sampling or the M-H algorithm to estimate the posterior distribution of the tuning parameter from the predefined grid of tuning parameter values. Since the resulting posterior distribution is discrete, it contains posterior probabilities for

each predefined tuning parameter value. Finally, we select the graphical model based on these posterior probabilities. Below we describe the A-R and the M-H algorithms used for this purpose.

2.2.1 Tuning parameter posterior simulation with the accept-rejection method

In A-R sampling, one generates samples from a density function f which is not any known probability density function and is difficult to simulate. If the functional form of f is known, one can actually propose simulated values from a candidate density g that is simpler to simulate. These values are either accepted or rejected with probability f/gM, where M is a constant satisfying $f/g \leq M$. The workflow of the A-R algorithm is outlined in Algorithm 1

Algorithm 1: Accept-Reject algorithm

input : Unknown target density f,
known candidate density g,
upper bound M;

output: Sample $\mathbf{x} = x[k]$ from the unknown target density f;

for $k = 1, 2, \ldots, K$ **do**

1. Generate $y \sim g$ and $U \sim Unif[0, 1]$;
2. Accept $x[k] = y$ if $U \leq f/gM$;
3. Return to 1 otherwise;

end

In the algorithm above, $Unif[0, 1]$ is the standard uniform distribution. For more details about A-R sampling, see, e.g. Robert and Casella (2010), pages 51–57.

In contrast to Algorithm 1, we do not propose tuning parameter values randomly from the entire support of the candidate density. Instead, we sample values from a grid of predefined tuning parameter values that we have already used to compute the solution path of GGMs. The sampling probability of each tuning parameter value is predetermined by the candidate density g. For example, if one uses a uniform distribution as the candidate density, each predefined tuning parameter value is proposed with equal probability.

In practice, the A-R method cannot be used to simulate posterior values of the tuning parameter from the conditional posterior distribution (4) because the

values of the posterior distribution can be extremely high due to exponentiation and the exponent in the posterior distribution (4). Choosing a suitable value for the parameter M is thus extremely hard and the implementation of the algorithm is impractical. We therefore decided to use the logarithm of the conditional posterior distribution because it does not change the support of the conditional posterior distribution and enables practical implementation of MCPeSe.

When we substitute the Glasso estimate of the precision matrix $\widehat{\Theta}$ into the conditional posterior (4) and ignore the normalizing constant C, the logarithm of the conditional posterior distribution multiplied with 2 is

$$2 \log p(\rho, \widehat{\Theta}|data) \propto p(p+1) \log(N\rho/2) - N\rho||\widehat{\Theta}||_1 + 2 \log p(\rho), \tag{5}$$

when using the parametrization $\lambda = N\rho$.

We formally describe the A-R algorithm used to simulate tuning parameter values from the log-posterior (5) in Algorithm 2.

Algorithm 2: A-R tuning parameter log-posterior simulation for Glasso

---

input : User-defined tuning parameter values on the grid: $\rho_1, \ldots, \rho_R$, corresponding Glasso estimates $\widehat{\Theta}(\rho_1), \ldots, \widehat{\Theta}(\rho_R)$ (the solution path),
candidate density g,
$M = \max \left\{ 2 \log p(\rho, \Theta|data) \mid \widehat{\Theta}(\rho_1), \ldots, \widehat{\Theta}(\rho_R) \right\}$;

output: Vector $\widehat{\boldsymbol{\rho}} = [\widehat{\rho}_k]$ with at most K elements;

for $k = 1, 2, \ldots, K$ **do**

1. Sample $\rho_{r'}$ from $g|\rho_1, \ldots, \rho_R$ and $U_M \sim Unif[0, Mg(\rho_{r'})]$;
2. Accept $\widehat{\rho}_k = \rho_{r'}$ if $U_M \leq 2 \log p(\rho_{r'}, \widehat{\Theta}(\rho_{r'})|data)$;
3. Return to 1 otherwise;

end

The greatest benefit of A-R sampling is its high computational speed. Its main drawback is that we can only generate samples from the log-posterior. Nevertheless, we should obtain a good representative sample of the tuning parameters simulated around the maximum (i.e., the mode) of the posterior distribution.

2.2.2 Tuning parameter posterior simulation with Metropolis–Hastings

Alternatively, the posterior sample of tuning parameter values can be generated using the M-H algorithm. Without going into excessive detail, we review the basic algorithm used to generate values from the target density f using the known candidate density g. The workflow of the M-H algorithm is outlined in Algorithm 3

Algorithm 3: Metropolis-Hastings algorithm

---

input : Unknown target density f,
known candidate density g,
initial value x_{old}, $x[1] = x_{old}$;

output: sample $\mathbf{x} = x[k]$ from the unknown target density f;

for $k = 1, \ldots, K$ **do**

1. Generate $x_{new} \sim g(x|x_{old})$;
2. Determine acceptance probability $d(x_{old}, x_{new}) = min\left\{1, \frac{f(x_{new})g(x_{old}|x_{new})}{f(x_{old})g(x_{new}|x_{old})}\right\}$ and generate $U \sim Unif[0, 1]$;
3. Accept $x[k+1] = x_{new}$ if $U \leq d(x_{old}, y)$, otherwise take $x[k+1] = x_{old}$;
4. Set $x_{old} = x[k+1]$;
5. Return to 1 otherwise;

end

For more information about the M-H algorithm, see, e.g., Robert and Casella (2010), pages 167–195.

The advantage of this approach compared to the A-R algorithm is that we can simulate tuning parameter values directly from the posterior (4) while using the logarithm of the conditional posterior multiplied by two (5) in the M-H algorithm acceptance probability. Therefore, the M-H algorithm can be used when the evaluated values of the posterior distribution are high. Moreover, different prior distributions for the tuning parameter can be considered by changing the logarithm of the tuning parameter prior distribution $p(\rho)$ in the log-posterior (5).

We formally describe the M-H algorithm with random walks that we use for

tuning parameter posterior estimation in Algorithm 4.

Algorithm 4: M-H tuning parameter posterior simulation for Glasso

---

input : User-defined tuning parameter values on the grid: $\rho_1, \ldots, \rho_R$,
corresponding Glasso estimates $\widehat{\Theta}(\rho_1), \ldots, \widehat{\Theta}(\rho_R)$ (the solution path),
initial value of ρ_{old} sampled from $\rho_1, \ldots, \rho_R$,
step length δ;

output: Vector $\widehat{\boldsymbol{\rho}} = [\widehat{\rho}_k]$ with K elements;

for $k = 1, 2, \ldots, K$ **do**

1. Choose ρ_{new} with random walk
(i) either as $\rho_{old-\delta}$ or $\rho_{old+\delta}$, $old - \delta \geq 1$, $old + \delta \leq R$
(ii) or with changing step length, $\rho_{new} \in \{\rho_1, \ldots, \rho_R\}$;
2. Calculate the logarithm of the acceptance probability
$\log d(\rho_{old}, \rho_{new}) =$
$\min\left\{0,\, 2\log p(\rho_{new}, \widehat{\Theta}(\rho_{new})|data) - 2\log p(\rho_{old}, \widehat{\Theta}(\rho_{old})|data)\right\}$
and generate $U \sim Unif[0, 1]$;
3. Accept $\widehat{\rho}_{k+1} = \rho_{new}$ if $\log U \leq \log d(\rho_{old}, \rho_{new})$, otherwise take $\widehat{\rho}_{k+1} = \rho_{old}$;
4. Set $\rho_{old} = \widehat{\rho}_{k+1}$;
5. Return to 1 otherwise;

end

Because we use random walks in the M-H algorithm, we can ignore the candidate density g in the acceptance probability. The disadvantages of the M-H algorithm compared to rejection sampling are that it is somewhat slower than the A-R method and depends on the choice of the step length δ. Nevertheless, our simulations indicate that using a doubly stochastic random walk proposal distribution where the step length is itself random in our M-H algorithm leads to fast-mixing Markov chains.

For now, our implementation of the M-H algorithm uses either a Uniform prior or a conjugate gamma prior restricted to the range $[\min(|\widehat{r}_{ij}|), \max(|\widehat{r}_{ij}|)]$. We emphasize that because we are using tuning parameter values from the predefined grid, the resulting posterior distribution is supported on a bounded interval. For example, the conditional posterior of Wang (2012) is a gamma distribution and there is no clear upper limit for the tuning parameter values whereas the support of our gamma posterior is $[\min(|\widehat{r}_{ij}|), \max(|\widehat{r}_{ij}|)]$.

Both of the A-R and the M-H algorithms used for tuning parameter posterior simulation share similarities with an empirical MC-EM type algorithm in that we combine sampling (the MC-step) with maximization (the EM-step or rather just the M-step). Formally, the workflow of MCPeSe is as follows:

1. Choose data-dependent tuning parameter values $\rho_r|Y$, $r = 1, \dots R$, where R is the number of grid points of predefined tuning parameter values.

2. Compute the *maximum a posterior* estimate (penalized MLE) of the precision matrix for each tuning parameter value $\widehat{\Theta}(\rho_r)$.

3. Pick additional data-dependent tuning parameter values $\rho_{r'}$ with probabilities determined by the candidate density g, $r^{'} \in \{1, \dots, R\}$ or use random walk.

4. If the sampled tuning parameter value and the corresponding precision matrix estimate $\widehat{\Theta}(\rho_{r'})$ satisfy the acceptance probability, take $\rho_{r'}$ as a sample from (4) or (5).

5. Return to 3. and repeat.

In particular, in the second step ("M-step"), the Glasso algorithm is used to maximize the precision matrix Θ at each value of the tuning parameter ρ.

We treat our parameter (the tuning parameter) as discrete, and strictly speaking it does not make sense to talk about a highest density interval (HDI) for a discrete parameter. However, we can compute an approximate 95% HDI for the discrete parameter that will still reflect the accuracy of estimation to at least some degree.

2.2.3 Graphical model selection using the simulated tuning parameter posterior probabilities

Although the algorithms we have introduced are used to estimate the posterior distribution of the tuning parameter, our goal is to select a point estimate for the undirected network computed with Glasso. Moreover, we want to select the GGM from the solution path to avoid unnecessary computation. To solve the graphical model selection problem, we get indirect information about the network solution path by inspecting the posterior probabilities of the corresponding

tuning parameter values. First we select the tuning parameter from the pre-specified grid, which is characteristic for the simulated posterior distribution. Then we select the estimated undirected network, which is computed using the selected tuning parameter value.

One can use either the posterior mean or the *maximum a posteriori* (MAP) of the estimated posterior distribution as a point estimate of the parameter of interest. However, the MAP estimate might not be fully representative of the whole posterior distribution. This non-representativeness could be particularly severe when the sample size is very small compared to the number of variables of interest.

We therefore favor the posterior mean over MAP estimation because it is usually more consistent with both the information gained from the observations and the prior assumptions. Moreover, having very similar posterior probabilities for most of regularization parameter candidates (which occurs when the posterior is seemingly flat) suggests that the corresponding network estimates might perform competitively in terms of graph learning. It is therefore reasonable to include all of this information in the point estimate.

We propose to select an estimate for the tuning parameter from the pre-specified grid whose corresponding value $\widehat{\rho}$ is the smallest among the tuning parameters values greater than or equal to the expected value of the simulated ρ values, that is

$$\widehat{\rho} = \sup\{\rho_r : \widehat{\rho}_r \geq \overline{\rho},\, r = 1, \dots R\} \tag{6}$$

where $\overline{\rho}$ is the mean of the posterior (log-posterior) of the tuning parameters sampled with the M-H (A-R) algorithm, respectively. Actually, $\overline{\rho}$ is the weighted mean estimate, which reflects information over the whole posterior distribution and implicitly from the whole network solution path. Choosing a graphical model corresponding to the first tuning parameter value above the expected value will reduce the number of edges in the selected network even though the posterior mean value is smaller than the MAP value in high dimensional settings. Of course, it is always helpful to inspect the estimated posterior distribution of the tuning parameter closely to gain indirect insights into all of the undirected network estimates.

In Fig 2 we illustrate the discrete posterior and empirical prior distributions

of the tuning parameter when the sample size N changes but the number of parameters P does not. When the sample size is very small compared to the number of variables (Fig 2 (A1)), the posterior distribution is left-skewed and the tuning parameter value corresponding to the MAP estimate is large. The corresponding graphical model would be very sparse, containing only a handful of edges. Nevertheless, this posterior distribution of the tuning parameter shows that a simple GGM should be selected. This is an intuitively reasonable outcome because there are several spurious edges in the estimated graph due to the meager information gained from just a few simulated observations. Fig 2 shows nicely that the shape of the posterior distribution of the tuning parameter changes when the sample size increases, which is desirable because when the sample size is extremely large the sample covariance matrix converges to the true covariance matrix due to the law of large numbers. We emphasize that sample sizes are not usually this large in high-dimensional data settings; we simply show these cases for illustrative purposes. If the sample size is very large compared to the number of variables ($N \gg P$), it might be reasonable to use "classical estimation methods" (MLE, hypothesis testing etc.) instead of Bayesian modeling (Williams and Rast, 2019; Wysocki and Rhemtulla, 2019).

In Fig 3 we compare the posterior distributions of the tuning parameter when they are estimated with both MCPeSe and the fully Bayesian Model (BGlasso). Both methods use the same hyperprior $\lambda \sim Gamma(1, 1/10)$ (Wang, 2012). In this example, we constructed the pre-specified grid of 100 ($R = 100$) tuning parameter values for MCPeSe based on the MCMC chain output of BGlasso to make the tuning parameter posteriors more comparable. This example shows that both MCPeSe and the fully Bayesian model produce very similar tuning parameter posterior distributions and the posterior means, assuming the support of the candidate density and the fully Bayesian model are the same. Otherwise the definition of the grid seems to have an effect on the shape of the posterior distribution.

2.3 Materials

2.3.1 Materials: simulated data

We generate artificial data to examine and illustrate the performance of MCPeSe. We consider five different network models in our simulations. Each model is

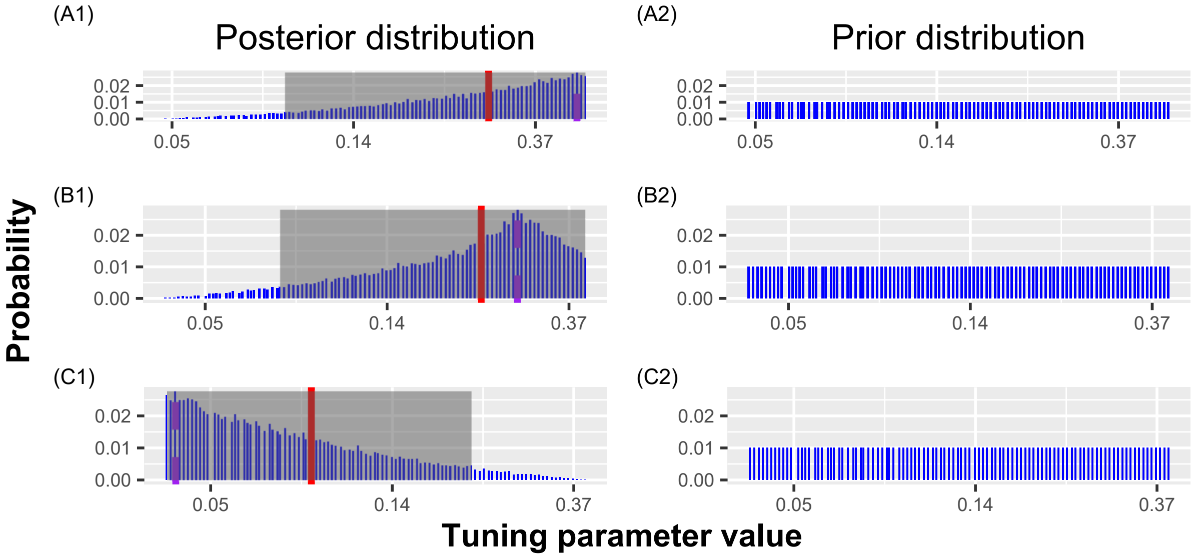

Figure 2: An illustration of the posterior distribution of tuning parameter values simulated using our M-H algorithm when $R = 100$ and $P = 500$ as the sample size changes: (**A1**, **A2**) $N = 200$; (**B1**, **B2**) $N = 4000$; (**C1**, **C2**) $N = 10^4$. In this example we use a Uniform prior $Unif[min(|\widehat{r}_{ij}|), max(|\widehat{r}_{ij}|)]$ and a double stochastic random walk with changing step length to generate MCMC samples of length 5×10^4 (with 1000 burn-in steps). For the posterior distribution, the approximate 95% highest density interval (dark gray area) is illustrated. The red solid line is the posterior mean estimate and the purple dashed line is the posterior mode (MAP estimate). The horizontal axis uses a log-scale. The GGM considered here is the Erdős-Rényi model (Random model) described in the Materials Section.

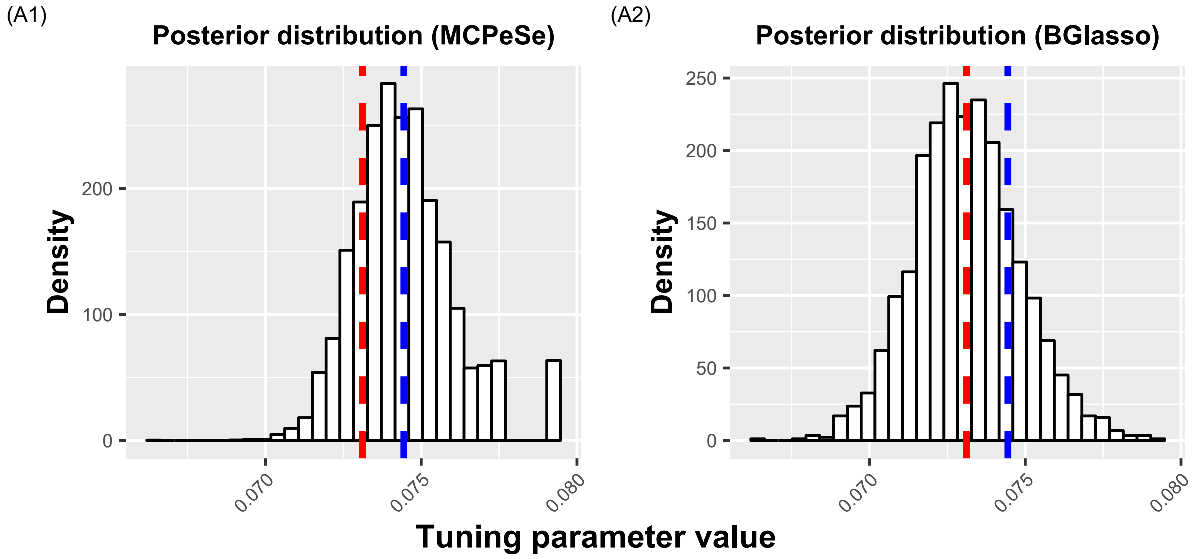

Figure 3: The posterior distributions estimates of tuning parameter values when $R = 100$, $P = 100$ and $N = 200$: (**A1**) MCPeSe output from 10^5 MCMC steps after 1000 burn-in steps using the M-H tuning parameter posterior simulation with a doubly stochastic random walk proposal distribution and gamma prior; (**A2**) BGlasso outputs from 2000 MCMC steps after 500 burn-in steps. The red dashed line is the posterior mean estimate of the BGlasso and the blue dashed line is the posterior mean estimate of MCPeSe. The GGM considered here is the Erdős-Rényi model (Random model) described in the Materials Section.

associated with the corresponding binary valued adjacency matrix $A = [a_{ij}]$, where the diagonal elements are zero, $a_{ii} = 0$, $i = 1, \ldots, P$. The following descriptions can also be found in the README file of the `huge` package:

1. *Band model (linear stepping stone model)*: The off-diagonal elements are set to $a_{ij} = 1$ if $|i - j| = 1$ and 0 otherwise. This produces a graph with $P - 1$ edges.

2. *The Barabási–Albert model (Scale-free model)*: The initial graph has two connected nodes and each new node is connected to only one node in the existing graph with a probability proportional to the degree of each node in the existing graph. This results in a graph with P edges.

3. *Cluster model*: The rows/columns are evenly partitioned into $P/20$ disjoint groups. Each pair of off-diagonal elements is set $a_{ij} = a_{ji} = 1$ for $i \neq j$ with probability 0.3 if both i and j belong to the same group and 0 otherwise. This results in about $3P/(P/20 - 1)$ edges in the graph.

4. *The Erdős-Rényi model (Random model)*: Each pair of off-diagonal elements is randomly set to $a_{ij} = a_{ji} = 1$ for $i \neq j$ with probability $P/3$, and 0 otherwise. This results in about $3(P - 1)/2$ edges in the graph.

5. *Hub model*: The rows/columns are evenly partitioned into $P/20$ disjoint groups. Each group is associated with a "center" row i in that group. Each pair of off-diagonal elements is set to $a_{ij} = a_{ji} = 1$ for $i \neq j$ if j also belongs to the same group as i and 0 otherwise. This results in $P - P/20$ edges in the graph.

Each model is simulated using the function "`huge.generator()`" from the R package `huge`, and we set $P = 500$ and $N = 200$ to mimic a high-dimensional case. We choose this sample size based on the recommendation of the StARS procedure: with at least 100 samples, the empirical and theoretical performance of StARS should be balanced (Liu et al., 2010).

2.3.2 Materials: empirical data

We use a real life high-dimensional data set to illustrate how MCPeSe can be used to find essential nodes in a huge network. We examine a data set concerning riboflavin (vitamin B_2) production in *Bacillus subtilis* (Lee et al., 2001; Zamboni

et al., 2005; Bühlmann et al., 2014). The data are longitudinal, but we analyze it as cross-section data as in Bühlmann et al. (2014).

This data set contains logarithm values representing gene expression levels of $P = 4088$ genes in $N = 71$ samples. The data set is publicly available in the R package `hdi`. Because of the extent of the problem at hand, we can only examine a couple of the most interesting genes in the terms of commonly used network characteristics. The conditional dependence relationships of these genes may be essential in riboflavin production.

2.4 Method comparison

2.4.1 Methods and comparison of diagnostics: simulated data

We illustrate how GGMs determined with MCPeSe compare to GGMs selected using other model selection criteria. In particular, we compare MCPeSe to three tuning parameter selection methods available in the `huge` R package: eBIC, StARS and RIC. We exclude BGlasso from the comparison because it would take several days to run BGlasso using only a moderate number of MCMC draws (see Fig 1).

First, we compute a solution path of GGMs with Glasso using $R = 50$ tuning parameter values. The maximal sparsity level (i.e. the proportion of present edges compared to the full graphical model) of the GGM estimates is restricted to 0.1, which is the default value used in the `huge` package. Each model selection method other than RIC selects the "optimal" GGM from the same solution path. When selecting the graphical model from the Glasso solution path with MCPeSe, we use a uniform prior distribution. We run the A-R algorithm 10^5 times to simulate the tuning parameter log-posterior utilizing a uniform density $Unif[\min(|\widehat{r}_{ij}|), \max(|\widehat{r}_{ij}|)]$ as the candidate distribution. We run the M-H algorithm 5×10^4 times (omitting 1000 burn-in steps) with the doubly stochastic random walk proposal distribution. In addition, we apply the selection rule (6) to select the point estimate for the tuning parameter with MCPeSe for both the A-R and the M-H methods. Both eBIC and StARS use an extra tuning parameter controlling the sparsity of the selected GGM: an additional tuning parameter γ in eBIC (Chen and Chen, 2008; Foygel and Drton, 2010) and a cut point value β in StARS (Liu et al., 2010). To mitigate the dependence of the model selection error on these extra parameters, we try multiple values

of both γ and β: $\gamma = 0, 0.005, 0.01, 0.05, 0.1, 0.5$ and $\beta = 0.05, 0.1$. After we have determined all possible γ, β and ρ combinations for eBIC and StARS, we select the model for each method that maximizes the Matthews Correlation Coefficient (MCC) as given below. We used MCC as the metric in eBIC and StARS hyperparameter selection because it measures the accuracy of prediction for both positive and negative classes of the confusion matrix. We believe that this approach maximizes the performance of these methods in practice and that the results obtained during their evaluation when applying this approach will not depend strongly on the choice of the extra hyperparameters γ and β. In real life, using MCC to select these extra parameters would not be practical. We note that when using RIC, a new tuning parameter value is determined based on random rotations; we use 20 random rotations in this case.

To determine how well the GGM selected with MCPeSe compares to those selected with other methods and to the ground truth, we compute the precision (Pre), sensitivity (also known as recall) (Sen), and Matthews Correlation Coefficient (MCC):

- Pre = $\frac{TP}{TP+FP}$
- Sen = $\frac{TP}{TP+FN}$
- MCC = $\frac{TP \times TN - FP \times FN}{\sqrt{(TP+FP)\times(TP+FN)\times(TN+FP)\times(TN+FN)}}$,

where TP is the number of true positives, FP is the number of false positives, FN is the number of false negatives and TN is the number of true negatives. Precision and sensitivity can vary between 0 and 1 while MCC can vary between -1 and 1 assuming no denominator equals zero. The closer that Pre, Sen, and MCC are to one, the better the performance of the graphical structure learning method. High precision but low sensitivity may indicate that the sparsity structure of the selected GGM is truthful but many important edges are left undiscovered. Conversely, low precision but high sensitivity may indicate that many correct edges in the selected GGM are found but the selected GGM is filled with spurious edges. The MCC is a correlation measure that considers all elements of the confusion matrix. It is particularly useful when the positive and negative classes of the confusion matrix are unbalanced, i.e. edge absence is more frequent than edge presence, as in the sparse network models considered here (Sedgewick et al., 2016). An MCC value close to one indicates

that true/false positives/negatives are correlated (in balance) compared to the ground truth model.

We select GGMs that maximize precision, sensitivity, and MCC (an Oracle model) simultaneously, and use them as reference points to illustrate the best possible performance of Glasso with respect to the simulated data. We note that it is rare for the selected GGMs to have both high precision and high sensitivity due to the great disparity between the sample size and number of parameter estimates.

As well as comparing the different methods, we show how MCPeSe captures the consistency of Glasso with simulated high-dimensional data when both P and N increase while the P/N ratio is held constant at 0.5. To this end, we set P to $100, 200, 300, 400, 500$ and 600 and use GGM selected with RIC as a point of comparison.

2.4.2 Methods and comparison diagnostics: empirical data

We compare MCPeSe with RIC because it produces GGMs with higher average precision in simulated data analysis and is a very time-efficient model selection method. We run the A-R algorithm 10^5 times and the M-H algorithm 5×10^4 times (with 1000 burn-in steps) with the doubly stochastic random walk proposal distribution to estimate the posterior distribution of the tuning parameter. We select the GGM whose corresponding tuning parameter value $\widehat{\rho}_r$ satisfies the condition (6). With RIC, we use 20 random rotations to choose the tuning parameter. Because of the size of the riboflavin data set, we only use a grid of $R = 20$ tuning parameter values for Glasso to construct the GGM solution path.

After constructing the GGM for the riboflavin data with Glasso and selecting the optimal graphs with both MCPeSe and RIC, we assess the importance of specific nodes in the network using the following centrality indices:

- node strength (degree), $\sum_j^P a_{ij}$, which is the number of adjacent edges of a given node i,
- closeness, $\sum_j^P 1/dist(i,j)$, which indicates the number of steps required to access every other node from a given node i,

- betweenness, $g_{jk}(i)/g_{jk}$ which is the number of shortest paths going through a given node i,

where a_{ij} is an element of the adjacency matrix $A = [a_{ij}]$, $dist(i,j)$ is the distance between node i and j, g_{jk} is the number of binary shortest paths between two nodes, and $g_{jk}(i)$ is the number of those paths going through node i. The higher the degree, closeness, and betweenness, the more important the node. The graph becomes increasingly disconnected as nodes with high betweenness are removed.

The definition of the closeness centrality index used here differs from the most common definition because high-dimensional networks constructed with Glasso are usually disconnected, which means that there will usually be at least two nodes in the estimated graph that are not connected to any other node (i.e. nodes with zero degree). The distance between two nodes with no path between them is infinite, making the conventional closeness measure not well-defined. We therefore evaluate the closeness of disconnected graphs in terms of the sum of inverted distances instead of the inverse sum of distances. The distance between nodes with no path between them thus becomes zero. This alternative definition of closeness for disconnected graphs was proposed by Opsahl et al. (2010).

3 Results

3.1 Simulated data results

Although we used a very sparse grid of tuning parameters (with only $R = 50$ different tuning parameter values), GGMs selected with MCPeSe are comparable to those obtained using other model selection methods in terms of precision, sensitivity, and MCC. In particular, the averaged MCC values for MCPeSe are close to the averaged Oracle values. On the whole, MCPeSe is more effective than other model selection methods in terms of both precision and sensitivity. We again note that it is very hard to select a GGM with both high precision and high sensitivity in high-dimensional cases: either one favors a simpler model (high precision) or a model with a close fit to the data (high sensitivity). Overall, the improvement in MCC results from an increase in sensitivity at the expense of precision. This may be undesirable in some biological applications.

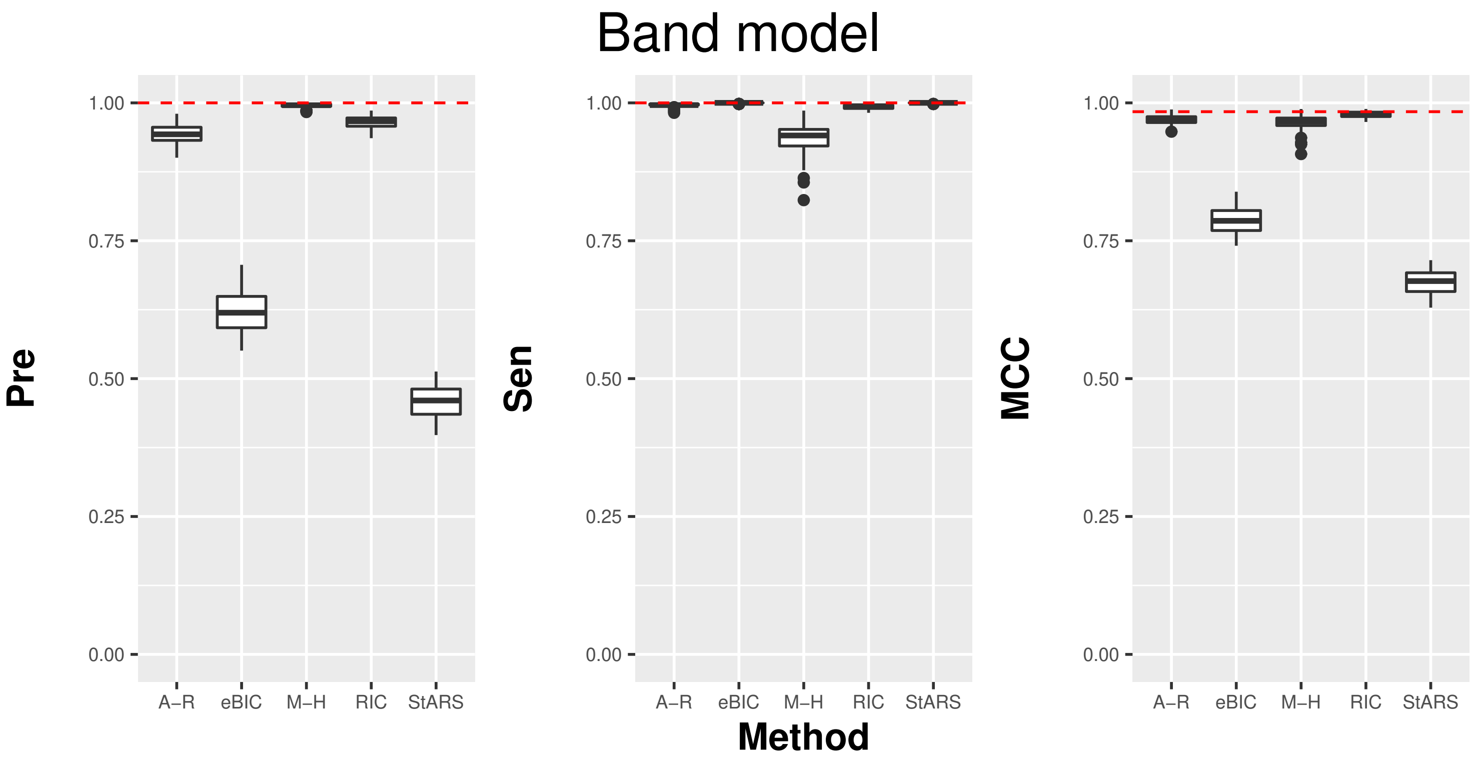

Figure 4: Binary classification test in terms of precision, sensitivity and Matthews correlation coefficient (MCC) for the **band** model based on 100 replications. The red dashed line indicates the best average value (median), which is chosen by inspecting each graphical model from the Glasso solution path (Oracle model).

Therefore, choosing an even larger tuning parameter value might help in such cases.

Estimation of the Barabási–Albert model (Scale-free model) seems to be very challenging with Glasso, and the performance of the graphical model selection methods is moderate in terms of the MCC (Fig 5). The L_1 penalty alone may be insufficient if the degree distribution of the unknown network follows a power law. Moreover, the sample size should be very large to recover the true scale-free network structure with high probability (Ravikumar et al., 2011). More structural information should thus be included if the undirected network of interest resembles the Barabási–Albert model (Schäfer and Strimmer, 2005; Liu and Ihler, 2011).

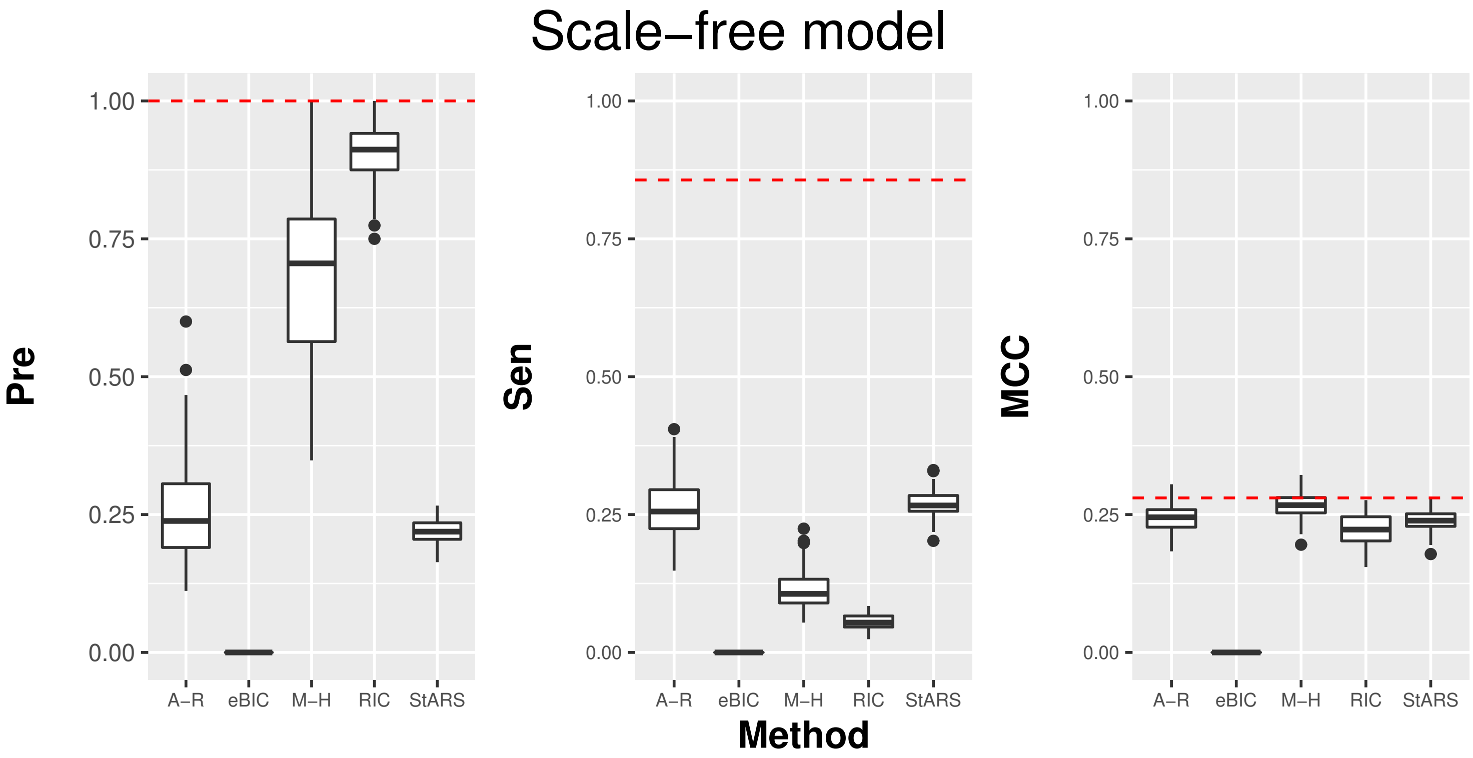

Figure 5: Binary classification test in terms of precision, sensitivity and Matthews correlation coefficient (MCC) for the **Scale-free** model based on 100 replications. The red dashed line indicates the best average value (median), which is chosen by inspecting each graphical model from the Glasso solution path (Oracle model).

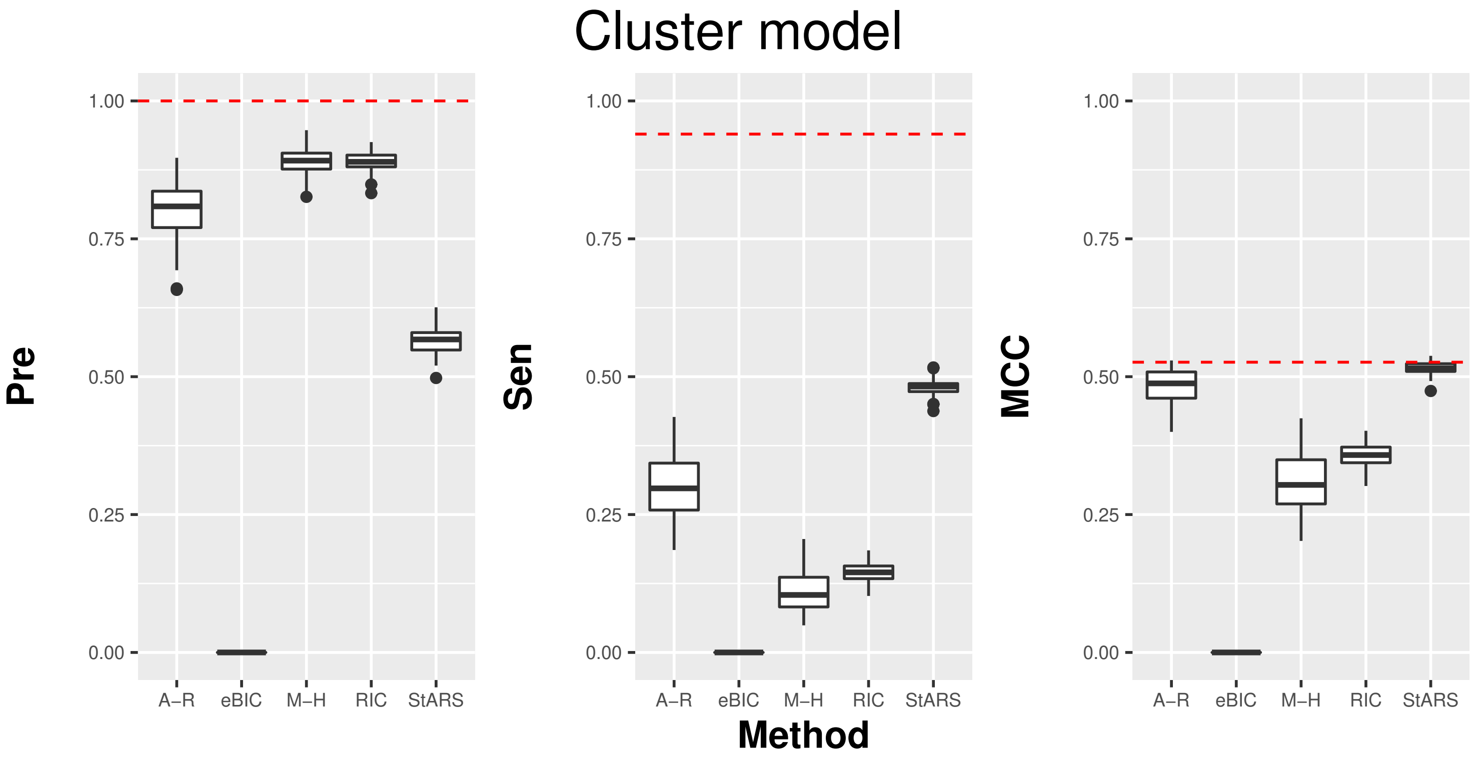

Figure 6: Binary classification test in terms of precision, sensitivity and Matthews correlation coefficient (MCC) for the **cluster** model based on 100 replications. The red dashed line indicates the best average value (median), which is chosen by inspecting each graphical model from the Glasso solution path (Oracle model).

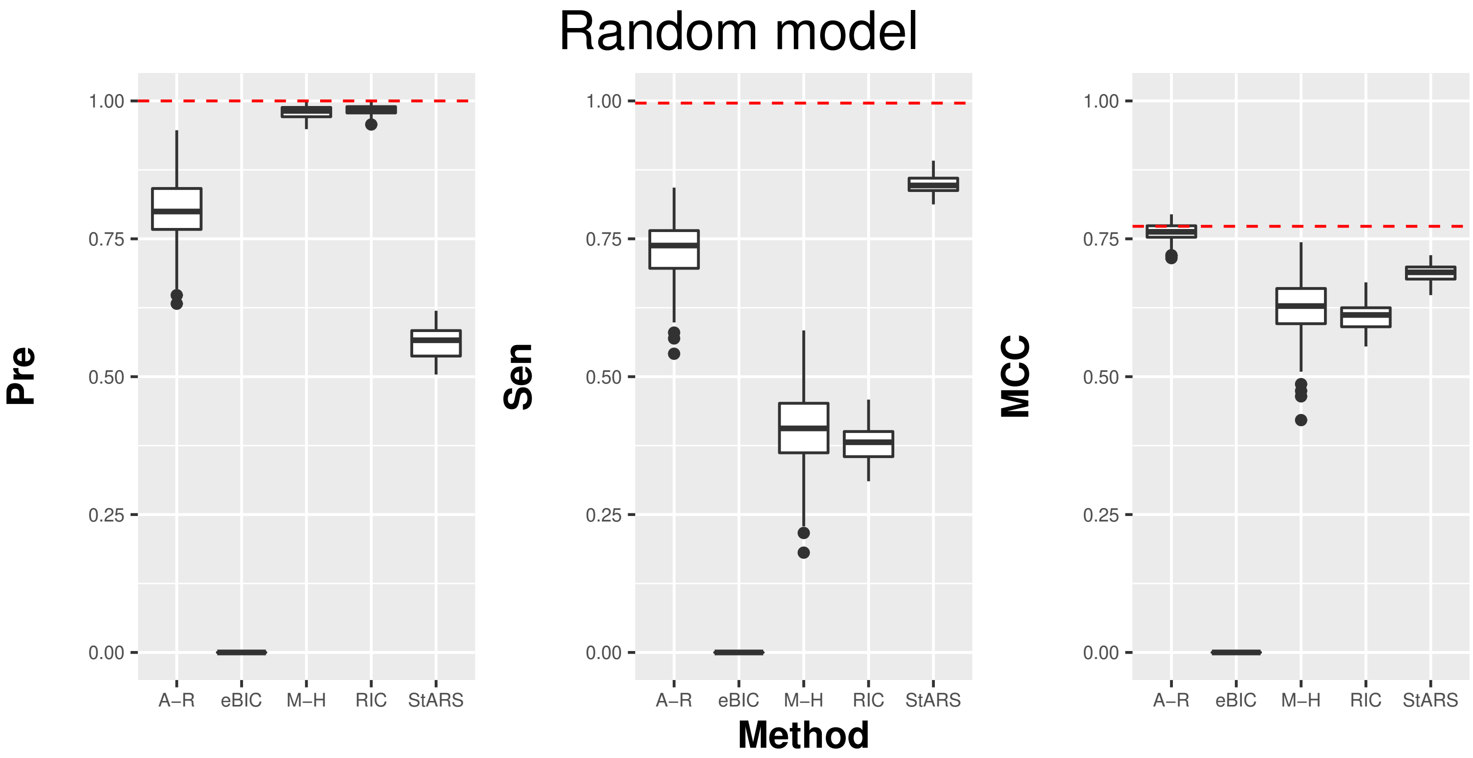

Figure 7: Binary classification test in terms of precision, sensitivity and Matthews correlation coefficient (MCC) for the **Erdős-Rényi** (random) model based on 100 replications. The red dashed line indicates the best average value (median), which is chosen by inspecting each graphical model from the Glasso solution path (Oracle model).

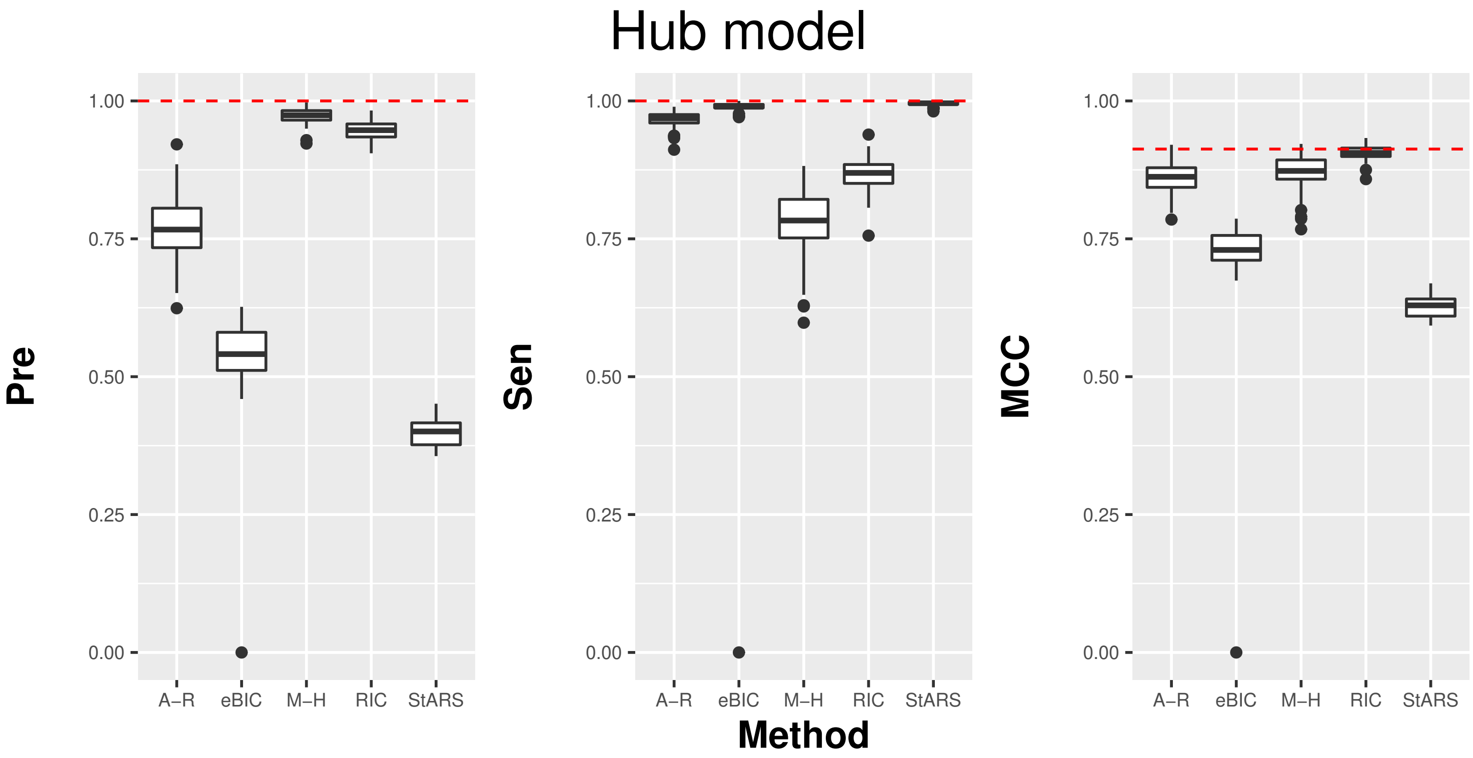

Figure 8: Binary classification test in terms of precision, sensitivity and Matthews correlation coefficient (MCC) for the **hub** model based on 100 replications. The red dashed line indicates the best average value (median), which is chosen by inspecting each graphical model from the Glasso solution path (Oracle model).

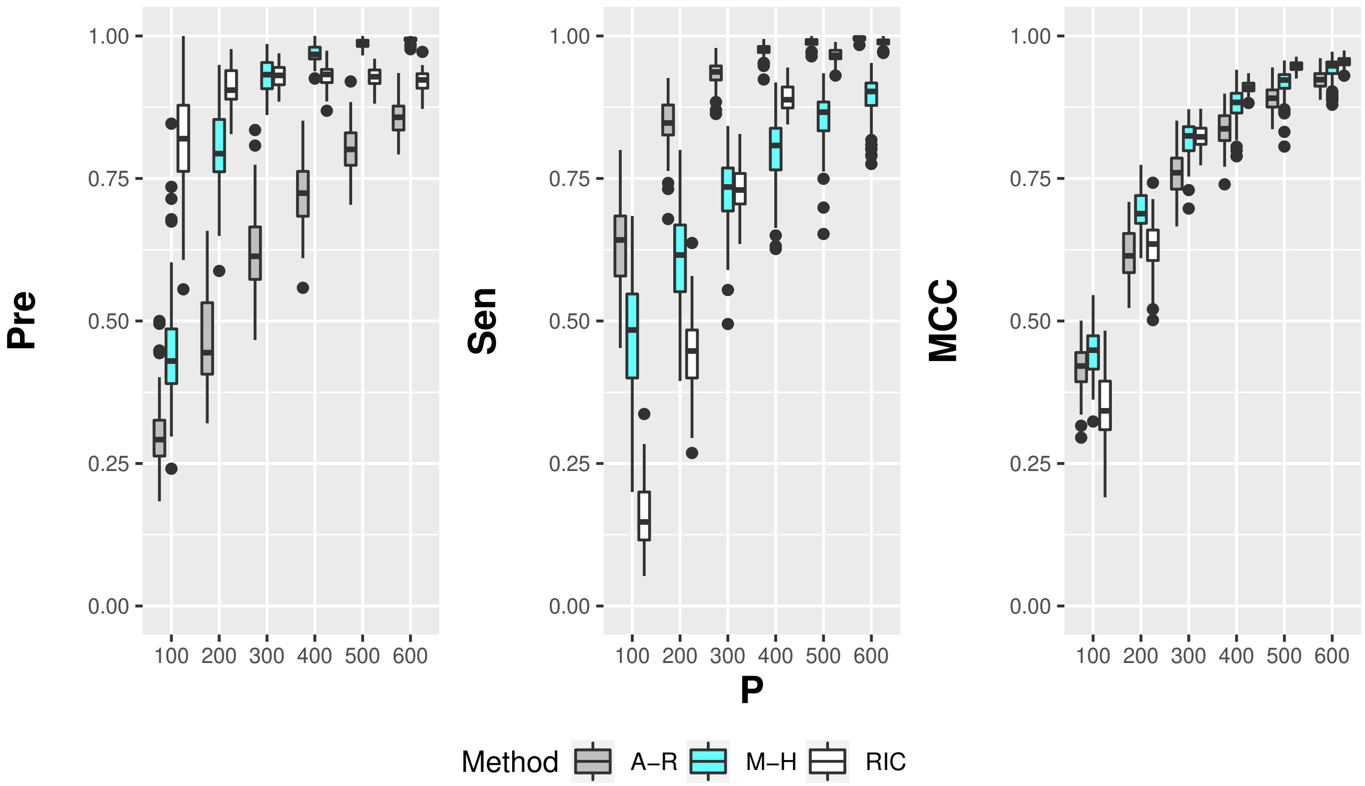

Figure 9: Binary classification test in terms of precision, sensitivity and Matthews correlation coefficient (MCC) when both the dimension P and the sample size N increase such that $N = \lfloor P/2 \rfloor$ for every value of P. We use RIC (empty boxplot) as a baseline for the accept-reject (A-R) and the Metropolis-Hastings (M-H) algorithms (filled boxplots). Classification measures are based on 100 replications. The graphical model considered here is the hub model.

When the number of non-zero entries of the precision matrix is at most $\mathcal{O}(P)$, all parameters that are zero are actually estimated as zero with probability tending to one when using Glasso (Lam and Fan, 2009). We choose the hub model to illustrate the consistency of MCPeSe because this graphical model clearly satisfies the condition mentioned earlier. We also determine graphical models with RIC and use it as a reference for our method. Fig 9 shows that MCPeSe also captures the high-dimensional consistency of Glasso (see also Ravikumar et al., 2011).

Finally, we illustrate how using a wider grid of tuning parameter values

changes the results. We show this using the Scale-free model because the performance of Glasso in terms of MCC was quite modest compared to other graphical models considered here. Fig 10 shows results obtained when using different numbers of tuning parameter values with Glasso. It seems that the overall performance of MCPeSe is consistent with the Oracle estimator in terms of MCC. The performance of Glasso is reduced in terms of precision, sensitivity, and MCC because of how the predefined tuning parameter sequence is defined in the `huge` package: grids used in Fig 10 are distinct due to the different number of grid points considered in the same interval. There is also a clear decline in the binary classification test results with respect to the Oracle estimates.

3.2 Empirical data results

After computing the solution path of 20 different GGMs with Glasso it still takes almost 12 minutes to select the optimal graph with RIC whereas running the A-R and M-H algorithms takes around one and seven seconds respectively with a standard desktop computer (64-bit operating system, 4 cores, and 32 GB RAM). The (log-) posterior distribution of the tuning parameter generated with the (A-R) M-H algorithm is left-skewed, resembling the shape of the posterior distribution plotted in Fig 2 (results not shown). Because it is hard to investigate all nodes of the riboflavin network ($P = 4088$), we only discuss here the five nodes with the highest values for the node strength, closeness and betweenness centrality measures. Genes corresponding to these nodes are listed in Fig 11.

As we predicted, all GGMs selected with MCPeSe and RIC are disconnected: the networks selected with the A-R algorithm, the M-H algorithm, and RIC break the graph into 2, 101, and 137 connected components, respectively. Considering the definition of the measures of centrality, the node in the largest component of the network usually produces the highest betweenness, closeness and strength values because it can reach more other nodes than a node in a smaller component. Therefore, these nodes/genes are most likely to play essential roles in the overall riboflavin co-expression network.

Four out of five of the nodes with the top five strength scores are identical in the networks estimated with MCPeSe and RIC. These nodes are the hub nodes of the network and are thus essential genes in the gene co-expression process. The gene “YMAE_at” has one of the five highest closeness centrality values

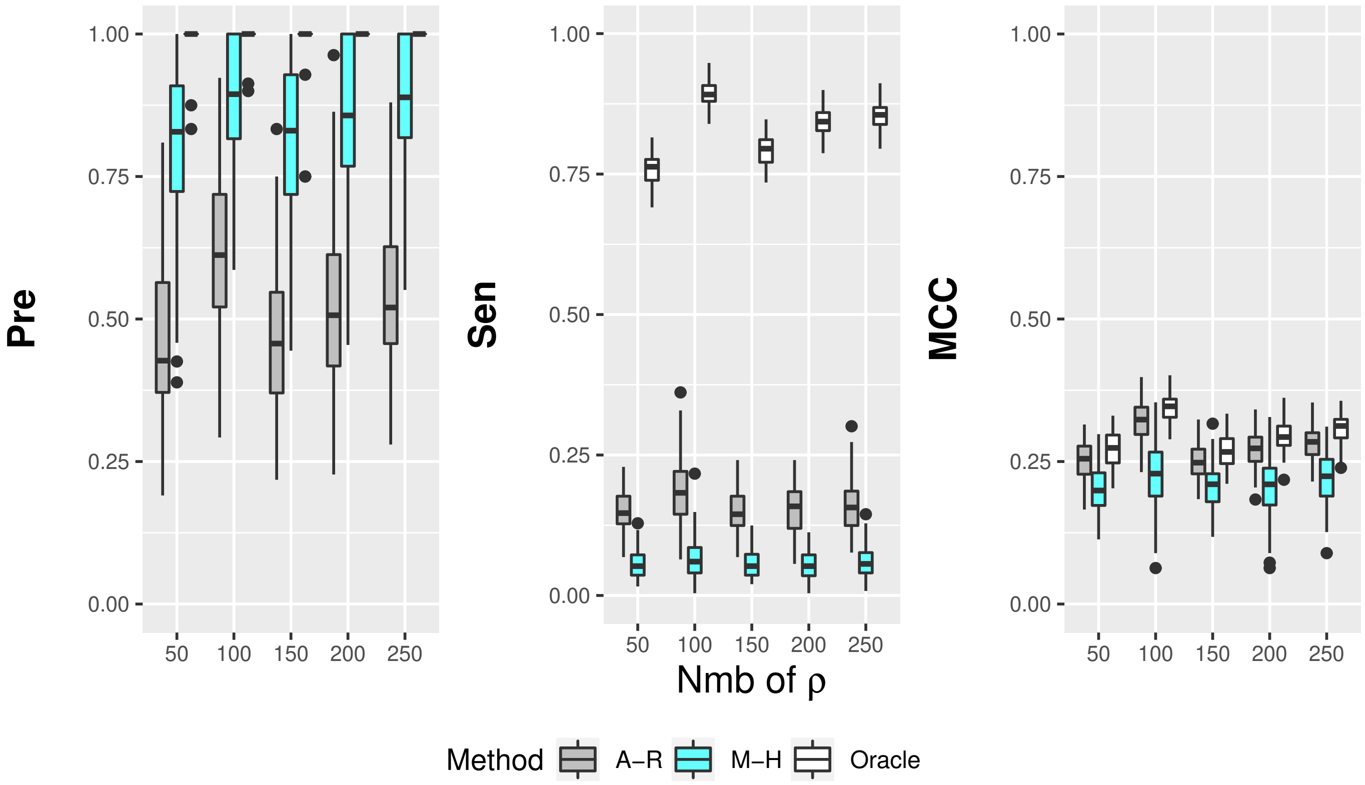

Figure 10: Binary classification test results in terms of precision, sensitivity and Matthews correlation coefficient (MCC) when the grid of the tuning parameter changes ("Nmb of ρ"). In this example, tuning parameters are plotted on a uniform (non-log) scale and $P = 250$ and $N = \lfloor P/2 \rfloor$. GGMs selected with the accept-reject (A-R) and the Metropolis-Hastings (M-H) algorithms are illustrated with filled boxplots and Oracle estimates with empty boxplots. These results are based on 100 simulation replications. The graphical model considered here is the Barabási–Albert model (Scale-free model).

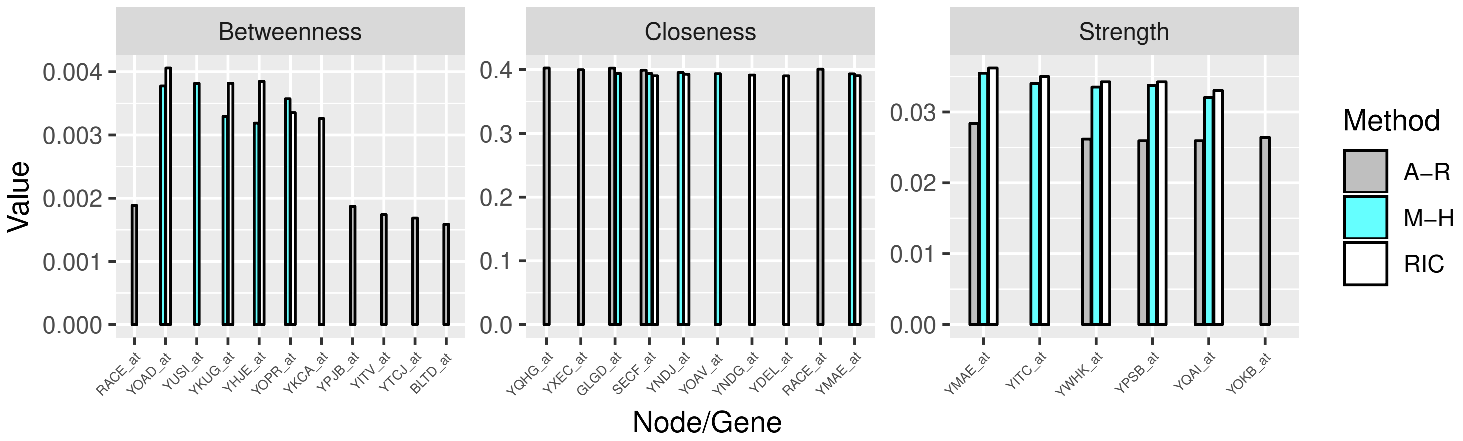

Figure 11: The five largest values of the normalized centrality measures of the riboflavin GGM determined with Glasso. The tuning parameter is selected using the accept-reject (A-R) and Metropolis-Hastings (M-H) algorithms as well as RIC.

in the GGM determined with MCPeSe. Because it is also the node with the highest strength centrality, we suspect that it plays a very important role in the gene co-expression of the riboflavin network based on this co-expression data.

4 Discussion

We have shown that an empirical Bayesian tuning parameter selection procedure that uses either the accept-rejection or the Metropolis-Hastings algorithm is a very efficient method for choosing a tuning parameter value for the Glasso algorithm.

The biggest problem when implementing MCPeSe is choosing the candidate distribution g and a proper upper limit M in the A-R algorithm 2 and the step length δ in the M-H algorithm 4. However, our choices of g and M (see Algorithm 2) seem to produce reasonable GGM estimates. Furthermore, the doubly stochastic random walk seems to be a reasonable choice for the M-H algorithm, leading to fast-mixing Markov chains. Another practical problem that may arise is that computing the whole solution path of high-dimensional networks requires multiple gigabytes of disk space and efficient machine learning algorithms. This can be a problem if one lacks access to cutting-edge computer

hardware.

In the future, it would be interesting to study how MCPeSe could be applied together with Bayesian model averaging so that the whole Glasso solution path information will be optimally utilized to estimate the precision matrix.

The MCPeSe R script, a demo script, and a collection of scripts used to prepare the material in this paper are publicly available at GitHub under the GPL license (https://github.com/markkukuismin/MCPeSe).

5 Conflict of interest

None declared.

References

Banerjee, O., Ghaoui, L. E., and d'Aspremont, A. (2008). Model selection through sparse maximum likelihood estimation for multivariate Gaussian or binary data. *Journal of Machine Learning Research*, 9:485–516.

Basu, S., Duren, W., Evans, C. R., Burant, C. F., Michailidis, G., and Karnovsky, A. (2017). Sparse network modeling and metscape-based visualization methods for the analysis of large-scale metabolomics data. *Bioinformatics*, 33:1545–1553. https://doi.org/10.1093/bioinformatics/btx012.

Bühlmann, P., Kalisch, M., and Meinshausen, N. (2014). High-dimensional statistics with a view toward application in biology. *Annual Review of Statistics and Its Application*, 1:255–278. https://doi.org/10.1146/annurev-statistics-022513-115545.

Bühlmann, P. and Van De Geer, S. (2011). *Statistics for High-Dimensional Data: Methods, Theory and Applications.* Springer Science & Business Media, London, UK.

Cai, T., Liu, W., and Luo, X. (2011). A constrained l_1 minimization approach to sparse precision matrix estimation. *Journal of the American Statistical Association*, 106:594–607. https://doi.org/10.1198/jasa.2011.tm10155.

Carvalho, C. M. and Scott, J. G. (2009). Objective Bayesian model selection in Gaussian graphical models. *Biometrika*, 96:497–512. https://doi.org/10.1093/biomet/asp017.

Casella, G. (2001). Empirical Bayes Gibbs sampling. *Biostatistics*, 2:485–500. https://doi.org/10.1093/biostatistics/2.4.485.

Casella, G., Robert, C. P., and Wells, M. T. (2004). Generalized accept-reject sampling schemes. *Lecture Notes-Monograph Series*, 45:342–347.

Chen, J. and Chen, Z. (2008). Extended Bayesian information criteria for model selection with large model spaces. *Biometrika*, 95:759–771. https://doi.org/10.1093/biomet/asn034.

Edwards, D. (2000). *Introduction to Graphical Modelling*. Springer, New York, USA, 2nd edition.

Epskamp, S. and Fried, E. I. (2018). A tutorial on regularized partial correlation networks. *Psychological Methods*, 23:617–634. http://dx.doi.org/10.1037/met0000167.

Fattahi, S. and Sojoudi, S. (2019). Graphical lasso and thresholding: Equivalence and closed-form solutions. *Journal of Machine Learning Research*, 20:1–44.

Fitch, A. M. and Jones, M. B. (2008). Shortest path analysis using partial correlations for classifying gene functions from gene expression data. *Bioinformatics*, 25:42–47. https://doi.org/10.1093/bioinformatics/btn574.

Foygel, R. and Drton, M. (2010). Extended Bayesian information criteria for Gaussian graphical models. In Lafferty, J., Williams, C., Shawe-Taylor, J., Zemel, R., and Culotta, A., editors, *Advances in Neural Information Processing Systems 23*, pages 604–612. Curran Associates, Inc., USA.

Friedman, J., Hastie, T., and Tibshirani, R. (2008). Sparse inverse covariance estimation with the graphical lasso. *Biostatistics*, 9:432–441. https://doi.org/10.1093/biostatistics/kxm045.

Friedman, J., Hastie, T., and Tibshirani, R. (2010). Regularization paths for generalized linear models via coordinate descent. *Journal of Statistical Software*, 33:1–22. http://dx.doi.org/10.18637/jss.v033.i01.

Khondker, Z. S., Zhu, H., Chu, H., Lin, W., and Ibrahim, J. G. (2013). The Bayesian covariance lasso. *Statistics and its Interface*, 6:243–259. http://dx.doi.org/10.4310/SII.2013.v6.n2.a8.

Krämer, N., Schäfer, J., and Boulesteix, A.-L. (2009). Regularized estimation of large-scale gene association networks using graphical Gaussian models. *BMC Bioinformatics*, 10:384. https://doi.org/10.1186/1471-2105-10-384.

Lam, C. and Fan, J. (2009). Sparsistency and rates of convergence in large covariance matrix estimation. *Annals of statistics*, 37:4254 – 4278. http://dx.doi.org/10.1214/09-AOS720.

Lee, J. D. and Hastie, T. J. (2015). Learning the structure of mixed graphical models. *Journal of Computational and Graphical Statistics*, 24:230–253. https://doi.org/10.1080/10618600.2014.900500.

Lee, J.-M., Zhang, S., Saha, S., Santa Anna, S., Jiang, C., and Perkins, J. (2001). RNA expression analysis using an antisense *Bacillus subtilis* genome array. *Journal of Bacteriology*, 183:7371–7380. https://doi.org/10.1128/JB.183.24.7371-7380.2001.

Li, Z., Bai, J., and Zhou, W. (2018). Learning Gaussian graphical models using discriminated hub graphical lasso. In *2018 IEEE International Conference on Acoustics, Speech and Signal Processing (ICASSP)*, pages 2471–2475. IEEE.

Liu, H., Roeder, K., and Wasserman, L. (2010). Stability approach to regularization selection (StARS) for high dimensional graphical models. In Lafferty, J. D., Williams, C. K. I., Shawe-Taylor, J., Zemel, R. S., and Culotta, A., editors, *Advances in Neural Information Processing Systems 23*, pages 1432–1440. Curran Associates, Inc., USA.

Liu, H. and Wang, L. (2017). TIGER: a tuning-insensitive approach for optimally estimating Gaussian graphical models. *Electronic Journal of Statistics*, 11:241–294. https://doi.org/10.1214/16-EJS1195.

Liu, Q. and Ihler, A. (2011). Learning scale free networks by reweighted l_1 regularization. In Gordon, G., Dunson, D., and Dudík, M., editors, *Proceedings of the Fourteenth International Conference on Artificial Intelligence and Statistics*, volume 15 of *Proceedings of Machine Learning Research*, pages 40–48, USA. PMLR.

Liu, W. and Luo, X. (2015). Fast and adaptive sparse precision matrix estimation in high dimensions. *Journal of Multivariate Analysis*, 135:153–162. https://doi.org/10.1016/j.jmva.2014.11.005.

Lysen, S. (2009). *Permuted inclusion criterion: a variable selection technique.* PhD thesis, University of Pennsylvania.

Mazumder, R. and Hastie, T. (2012). Exact covariance thresholding into connected components for large-scale graphical lasso. *Journal of Machine Learning Research*, 13:723–736.

Meinshausen, N. and Bühlmann, P. (2006). High-dimensional graphs and variable selection with the LASSO. *The Annals of Statistics*, 34:1436–1462. https://doi.org/10.1214/009053606000000281.

Meinshausen, N. and Bühlmann, P. (2010). Stability selection. *Journal of the Royal Statistical Society: Series B (Statistical Methodology)*, 72:417–473. https://doi.org/10.1111/j.1467-9868.2010.00740.x.

Mestres, A. C., Bochkina, N., and Mayer, C. (2018). Selection of the regularization parameter in graphical models using network characteristics. *Journal of Computational and Graphical Statistics*, 27:323–333. https://doi.org/10.1080/10618600.2017.1366910.

Mohammadi, A. and Wit, E. C. (2015). Bayesian structure learning in sparse Gaussian graphical models. *Bayesian Analysis*, 10:109–138. http://dx.doi.org/10.1214/14-BA889.

Opsahl, T., Agneessens, F., and Skvoretz, J. (2010). Node centrality in weighted networks: Generalizing degree and shortest paths. *Social Networks*, 32:245 – 251. https://doi.org/10.1016/j.socnet.2010.03.006.

Petralia, F., Wang, L., Peng, J., Yan, A., Zhu, J., and Wang, P. (2018). A new method for constructing tumor specific gene co-expression networks based on samples with tumor purity heterogeneity. *Bioinformatics*, 34:i528–i536. https://doi.org/10.1093/bioinformatics/bty280.

Ravikumar, P., Wainwright, M. J., Raskutti, G., Yu, B., et al. (2011). High-dimensional covariance estimation by minimizing l_1-penalized log-

determinant divergence. *Electronic Journal of Statistics*, 5:935–980. http://dx.doi.org/10.1214/11-EJS631.

Robert, C. P. and Casella, G. (2010). *Introducing Monte Carlo Methods with R*, volume 18. Springer.

Schäfer, J. and Strimmer, K. (2005). A shrinkage approach to large-scale covariance matrix estimation and implications for functional genomics. *Statistical Applications in Genetics and Molecular Biology*, 4(1):–. https://doi.org/10.2202/1544-6115.1175.

Sedgewick, A. J., Shi, I., Donovan, R. M., and Benos, P. V. (2016). Learning mixed graphical models with separate sparsity parameters and stability-based model selection. *BMC bioinformatics*, 17(S5):S175. https://doi.org/10.1186/s12859-016-1039-0.

Wang, H. (2012). Bayesian graphical lasso models and efficient posterior computation. *Bayesian Analysis*, 7:867–886. https://doi.org/10.1214/12-BA729.

Wen, F., Chu, L., Liu, P., and Qiu, R. C. (2018). A survey on nonconvex regularization-based sparse and low-rank recovery in signal processing, statistics, and machine learning. *IEEE Access*, 6:69883–69906. https://doi.org/10.1109/ACCESS.2018.2880454.

Williams, D. R., Piironen, J., Vehtari, A., and Rast, P. (2018). Bayesian estimation of Gaussian graphical models with predictive covariance selection. *arXiv preprint arXiv:1801.05725*.

Williams, D. R. and Rast, P. (2019). Back to the basics: Rethinking partial correlation network methodology. *British Journal of Mathematical and Statistical Psychology*, n/a:n/a. https://doi.org/10.1111/bmsp.12173.

Wysocki, A. C. and Rhemtulla, M. (2019). On penalty parameter selection for estimating network models. *Multivariate Behavioral Research*, 0(0):1–15. https://doi.org/10.1080/00273171.2019.1672516.

Zamboni, N., Fischer, E., Muffler, A., Wyss, M., Hohmann, H.-P., and Sauer, U. (2005). Transient expression and flux changes during a shift from high to low riboflavin production in continuous cultures of *Bacillus subtilis. Biotechnology and Bioengineering*, 89:219–232. https://doi.org/10.1002/bit.20338.
